# Effects of antenatal hypnosis on maternal salivary cortisol during childbirth and six weeks postpartum—A randomized controlled trial

**DOI:** 10.1371/journal.pone.0230704

**Published:** 2020-05-01

**Authors:** Anette Werner, Chunsen Wu, Robert Zachariae, Ellen A. Nohr, Niels Uldbjerg, Åse Marie Hansen

**Affiliations:** 1 Institute of Clinical Research, Research Unit of Gynecology and Obstetrics, University of Southern Denmark, Odense, Denmark; 2 Department of Gynecology and Obstetrics, Odense University Hospital, Odense, Denmark; 3 Department of Oncology, Unit for Psychooncology and Health Psychology, Aarhus University Hospital, Aarhus, Denmark; 4 Department of Psychology, Aarhus University, Aarhus, Denmark; 5 Department of Gynecology and Obstetrics, Aarhus University Hospital Skejby, Aarhus, Denmark; 6 Department of Public Health, University of Copenhagen, Copenhagen, Denmark; 7 National Research Centre for the Working Environment, Copenhagen, Denmark; La Sapienza University of Rome, ITALY

## Abstract

**Background:**

Cortisol has been used to capture psychophysiological stress during childbirth and postpartum wellbeing. We explored the effect of a brief antenatal training course in self-hypnosis on salivary cortisol during childbirth and 6 weeks postpartum.

**Methods:**

In a randomized, controlled trial conducted at Aarhus University Hospital Skejby Denmark during the period January 2010 until October 2010, a total of 349 healthy nulliparous women were included. They were randomly allocated to *a hypnosis group* (n = 136) receiving three one-hour lessons in self-hypnosis with additional audio-recordings, *a relaxation group* (n = 134) receiving three one-hour lessons in various relaxation methods with audio-recordings for additional training, and *a usual care group* (n = 79) receiving ordinary antenatal care only. Salivary cortisol samples were collected during childbirth (at the beginning of the pushing state, 30 minutes, and 2 hours after childbirth), and 6 weeks postpartum (at wake up, 30 minutes after wake up, and evening). Cortisol concentrations were compared using a linear mixed-effects model. Correlations between cortisol concentrations and length of birth, experienced pain and calmness during birth were examined by a Spearman rank correlation test.

**Findings:**

During childbirth, week correlations were found between cortisol concentrations 30 minutes after childbirth and length of birth. In the beginning of the pushing state and 2 hours after childbirth, we found a tendency towards higher cortisol concentrations in the hypnosis group compared to the other two groups (hypnosis versus relaxation p = 0.02 and 0.03, hypnosis versus usual care p = 0.08 and 0.05). No differences were observed in cortisol concentrations between the groups 30 minutes after childbirth (hypnosis versus relaxation p = 0.08, hypnosis versus usual care 0.10) or 6 weeks postpartum (hypnosis versus relaxation: p = 0.85, 0.51, and 0.68, hypnosis versus usual care: p = 0.85, 0.93, and 0.96).

**Conclusion:**

Antenatal hypnosis training may increase the release of cortisol during childbirth with no long-term consequences. Further research is needed to help interpret these findings.

## Introduction

Childbirth is a time of intense psychophysiological stress. Supporting the woman in an adequate way is important since negative birth experiences have been associated with both short-term and long-term consequences, including postpartum depression [1, 2] and childbirth-related post-traumatic stress [3, 4].

Several studies have used cortisol as a biomarker for stress response during childbirth [5] as well as a biomarker to capture postnatal mood disorders [6]. Cortisol is a steroid hormone regulated by a negative feedback process through the hypothalamic-pituitary-adrenal (HPA) axis. It is released in the adrenal cortex in response to a perceived challenge, and once the challenge is over, the body downregulates the response. If the stress is repeated or sustained, it can over time result in a failure to down and upregulate this response [7]. A primary function of cortisol is to provide energy by playing a role in the metabolism of glucose, proteins and lipids [5]. Cortisol has a diurnal variation with levels peaking in the morning, prior to awakening, and decreasing progressively during the day to reach low levels in the evening [7]. During pregnancy, the diurnal variation of cortisol levels is maintained, but circulating cortisol levels increase. In late pregnancy, maternal cortisol levels reach concentrations twice as high as in non-pregnant subjects [8–10].

Maternal cortisol levels increase markedly from the onset of spontaneous labor, rise continuously during labor and delivery, and seem important for facilitating labor progress as well as maternal and fetal well-being during birth primarily by maintaining glucose equilibrium [5, 11, 12]. It has been suggested that maternal cortisol during labor is affected by psychophysiological stress as cortisol levels increase due to fear [5, 13, 14] experienced pain [15] and instrumental delivery [13, 16–18]. Cortisol levels also decrease with use of epidural analgesia which is the most effective form of pain relief for labor [13, 14].

In the postpartum period, the HPA axis will gradually return to pre-pregnant state. In most women, basal cortisol levels recover from the activated state within 2 weeks after childbirth [11, 19, 20]. Several studies have linked postnatal mood disorders to cortisol [21–24]. However, the findings are inconsistent as other studies did not find a correlation between cortisol and postnatal mood disorders [25–27]. The same remains for the correlation between psychological wellbeing in pregnancy and cortisol as some studies did confirm a correlation [28–30] and other studies failed to show a correlation [31–33]

Hypnosis has been used as an intervention to ease childbirth [34–37] and it may reduce the use of analgesia during labor [34]. We developed a brief course in antenatal self-hypnosis to provide women with skills to cope with childbirth. While we did not find any effects on use of epidural analgesia, pain experience and other obstetric birth outcomes [38, 39], a positive effect was found on the childbirth experience [40]. In the present report, we describe the effects of the intervention on the saliva cortisol response. We hypothesized that a brief course in antenatal self-hypnosis compared to a brief course in relaxation and usual care would help the woman to a) experience less emotional stress resulting in lower levels of cortisol during childbirth, and b) return to a normal daily cortisol response 6 weeks postpartum.

## Methods

### Design

The study was conducted at the Obstetrics Department at Aarhus University Hospital, Denmark. The trial was randomized, controlled, and single-blinded with a 3-arm group design. The primary outcomes of the trial were use of epidural analgesia and self-reported pain during delivery and have previously been reported [38]. The effect on salivary cortisol, reported in this paper, was a secondary pre-specified outcome.

### Ethical approval and registration

This trial was approved on December 15, 2008 by the Scientific Ethical Committee for the Region of Central Jutland, nr.M-200080200 in Denmark and by the Danish Data Protection Agency on November 5, 2008, nr.2088-41-2797. The trial was also reported to ClinicalTrials.gov, number NCT00914082.

### Population

All healthy women with uncomplicated singleton pregnancy referred to the hospital for childbirth were invited to join the study. During the period January 2010 until October 2010, all participants in the main study were additionally invited to collect salivary cortisol samples if they had signed up for the trial before their 32nd gestational week. In the three groups, participation rates in any saliva sampling were 97.8%, 98.5%, and 90.8%.

### Randomization and intervention

The participants were randomly allocated to either a hypnosis group (n = 139), an active comparison group (named “the relaxation” in the following) (n = 136), or a usual care control group (n = 87) using a computer generated interactive voice-response telephone randomization system which has previously been described in detail [38].

The women in the hypnosis group attended, alone without a partner, three 1-hour classes on self-hypnosis for childbirth held over three consecutive weeks with four supplementary audio recordings, including a 20-minute section especially meant for labor. The first class took place about gestational week 35.

The women in the relaxation group had three antenatal classes in various relaxation methods, each lasting one hour held over three consecutive weeks. They also attended the classes alone without a partner. As for the hypnosis intervention, the first class took place about gestational week 35 and the course also included audio recordings for homework and labor.

The usual care group received only ordinary antenatal care, which included a nuchal translucency scan about gestational week 12, an anomaly scan about gestational week 19, four to five visits at the midwifery clinic, and a tour of the birth department.

The interventions have previously been described in detail. We also reported a high compliance to the interventions, 97.8% in the hypnosis group and 96.4% in the relaxation group [38].

### Blinding

The midwives assisting the birth were blinded to the participant’s allocated treatment. Data management was performed without knowledge about the participant’s allocated treatment.

### Data collection and outcome

Baseline information of the participants derived from a web-based questionnaire completed by the participants at recruitment. This questionnaire also included the WHO-5 well-being index score, ranging from 0 to 100 with highest scores indicating positive well-being [41, 42].

A second questionnaire completed six weeks postpartum provided information about breastfeeding as well as childbirth- and pain experiences. We asked the women to what extent they had remained calm during labor and childbirth (perceived calmness) and how intense their labor pain had been at the end of the active phase and during the pushing phase. Responses were recorded on 11-point Likert scales ranging from 0 (nothing at all) to 10 (worst imaginable).

Information about the childbirth came from the database of “The Aarhus Birth Cohort”. Length of birth was for vaginal birth defined as “time from arriving at the birth department until the beginning of the pushing phase of second stage of labor” and “duration of the pushing phase of second stage of labor”. For emergency cesarean sections, it was defined as “duration from admission at the birth department until delivery”. In case of missing information or if the woman gave birth at another hospital, the necessary data were obtained from medical records.

### Saliva sampling

The women were asked to collect saliva for measurement of cortisol during pregnancy at gestational week 32, during childbirth, and 6 weeks postpartum. Saliva samples were collected in tubes (Salivette®).

During pregnancy and postpartum, the participants collected a sample at awakening, 30 minutes after awakening and in the evening at approximately 8 pm. A sample kit including 3 Salivette® tubes and written instructions on the sampling procedure were mailed to the participants approximately one week before they were supposed to collect the samples.

During childbirth, the midwife was instructed to collect three samples. The first sample was collected in the beginning of the pushing phase, and the second sample approximately 20–30 minutes after the childbirth. In case of perineal repair after childbirth, the second sample was collected after this and before the child started to breastfeed. The third sample was collected approximately 2 hours postpartum before routine care. If the woman delivered by caesarean section, the first sample was collected right before she entered the operating theater. The second sample was collected after the operation when the women had arrived at the recovery room and before the child started to breastfeed.

For all samples, the instruction was to avoid tooth brushing, food and drink 30 minutes prior to collecting the sample. The exact time of sampling should carefully be filled in on the vial when collecting each saliva sample. Samples were kept at 5°C and posted as soon as possible to the laboratory where they were stored at -20°C until analyzed.

Cortisol awakening response (CAR) was defined as the difference in cortisol concentration in nmol/l between the sample at awakening and the second sample 30 minutes after awakening per 30 minutes. Cortisol decline over the day (CDD) was defined as the difference in nmol/l between the highest morning cortisol concentration and the cortisol concentration in the evening sample at 8 PM per 1 hour.

### Hormonal analysis

The determination of cortisol in saliva was carried out with a competitive radioimmunoassay (RIA) designed for quantitative in vitro measurement of cortisol in serum, plasma, urine and saliva, the Spectria Cortisol Coated Tube RIA (Orion Diagnostica, Espoo, Finland). The sample volume was 150 μl, the range of the standard solutions prepared was 1.0−100.0 nmol/l, and the incubation time was 30 minutes at 37°C.

A method evaluation of certified reference material in water showed no bias of the methods. The recovery was 97% (95% CI: 94.0–100.9). The limit of detection (LOD) was 1.59 nmol/l. Between-run coefficients of variation (CVs) were 19% at 11.5 nmol/l and 16% at 49.2 nmol/l [43]. Concentrations below the LOD were assigned a random value between 0 and LOD extracted from a uniform distribution. Concentrations above 100 nmol/l were considered outliers and deleted from the sample.

To show equivalence between different runs, natural saliva samples (4.93 nmol/l and 25.8 nmol/l) were used as control materials and analyzed together with the samples. Westgard control charts were used to document that the trueness and the precision of the analytical methods remained stable [44]. The performance of the methods has been further validated by participation in interlaboratory comparison schemes [43, 45]

### Statistical power

The statistical power calculations in the main study were based on the primary outcome, use of epidural analgesia [38]. In total, we had a sample of 1222 participants. We planned to do an explorative study on cortisol and aimed at collecting saliva samples from 100 women in the hypnosis group, 100 in the active comparison group and 60 in the usual care group. As we expected that some participants would drop out, we invited 139 women from the hypnosis group, 136 from the active comparison group and 87 from the usual care group to this part of the study.

### Statistical analysis

Participant characteristics are presented as means with standard deviations for continuous variables and as frequencies for categorical variables.

Saliva cortisol concentrations, CAR and CDD are presented as means with standard deviations.

All statistical analysis was performed on the log-transformed cortisol concentrations since the distributions of these concentrations were skewed. A linear mixed effects model with autoregressive structure was used to estimate the differences in cortisol concentrations levels between the randomized groups. The hypnosis group was used as reference. Subsequently, the estimated differences in log-scale were transformed back to the original scale. As differences on a log-scale are corresponding to ratios on the original scale, the results are presented as a ratio between groups.

Intention to treat analysis was performed. In a second analysis, we adjusted for pre-defined potential confounders. We corrected for multiple hypotheses testing using the Holm-Bonferroni method.

To identify potential outliners, we used a QQ plot of the log-transformed cortisol concentrations. The outliners were then removed, and the analysis were repeated.

For pre-intervention data obtained during pregnancy, we assumed that the cortisol concentrations were different across the three time points. Therefore, the crude model included both randomization group, the time point (1: wake up, 2: + 30 min after wake up, and 3: evening, respectively), and an interaction term between the two variables, as well as time of sampling as a linear and squared function. The estimates were adjusted for the pre-specified potential confounders, smoking, season of birth, BMI and mother’s age, wellbeing at baseline and education.

For data obtained during childbirth, we also assumed that the effects on cortisol concentrations were different across the three time points. The crude model included randomization group, the time point (1:at the beginning of the pushing phase, 2: 30 minutes after childbirth, 3: two hours after childbirth), an interaction term between the two variables, and time of sampling as a linear and squared function. As saliva cortisol concentrations differed between the groups at baseline, we also added the log transformed cortisol concentration from the wake up sample and CAR in pregnancy. Intention to treat analysis was performed. In a second analysis, we excluded participants giving birth by planned cesarean section. Then we included use of epidural analgesia and mode of delivery in the model as well and adjusted for the pre-specified potential mentioned above and length of birth as well as additional antenatal training offered by private providers.

To account for missing values on cortisol samples during childbirth, sensitivity analysis was performed using multiple imputation. In this analysis, we excluded participants missing pre-intervention data obtained during pregnancy (cortisol samples at wake up and 30 minutes after wake up). Values for the missing cortisol samples were imputed using chained equations under the assumption of missing at random. Based on our best knowledge, the randomization group, mode of delivery, use of epidural analgesia, length of birth, and BMI were included in the imputation model. Predictive mean matching was used to impute cortisol concentration on the log-scale. A total of 1000 copies of the missing values were imputed.

The statistical model used for the cortisol concentrations in pregnancy was also used for the analysis 6 weeks after childbirth. However, in order to take the women’s baseline cortisol concentrations into account, we also added the cortisol concentration at wake up and CAR in pregnancy to the model. We adjusted for prespecified potential confounders, including season of birth, smoking, BMI, mother’s age, well-being at baseline, education and breastfeeding.

We used a Spearman rank correlation test to examine the correlation between saliva cortisol concentrations and psychological outcomes in the total sample. For pre-intervention data, the correlation between wellbeing at baseline and the cortisol concentration at wake up, the log-transformed CAR, and the log-transformed CDD, respectively, were tested. During childbirth, we explored the correlation between the cortisol concentration and experienced pain and between the cortisol concentration and calmness. We also explored the correlation between the cortisol concentration and length of birth. We repeated these analyses stratified by mode of delivery and use of epidural analgesia, respectively.

P- values below 0.05 were considered statistically significant. STATA version 15 was used for the statistical analysis.

## Results

**Fig 1:**
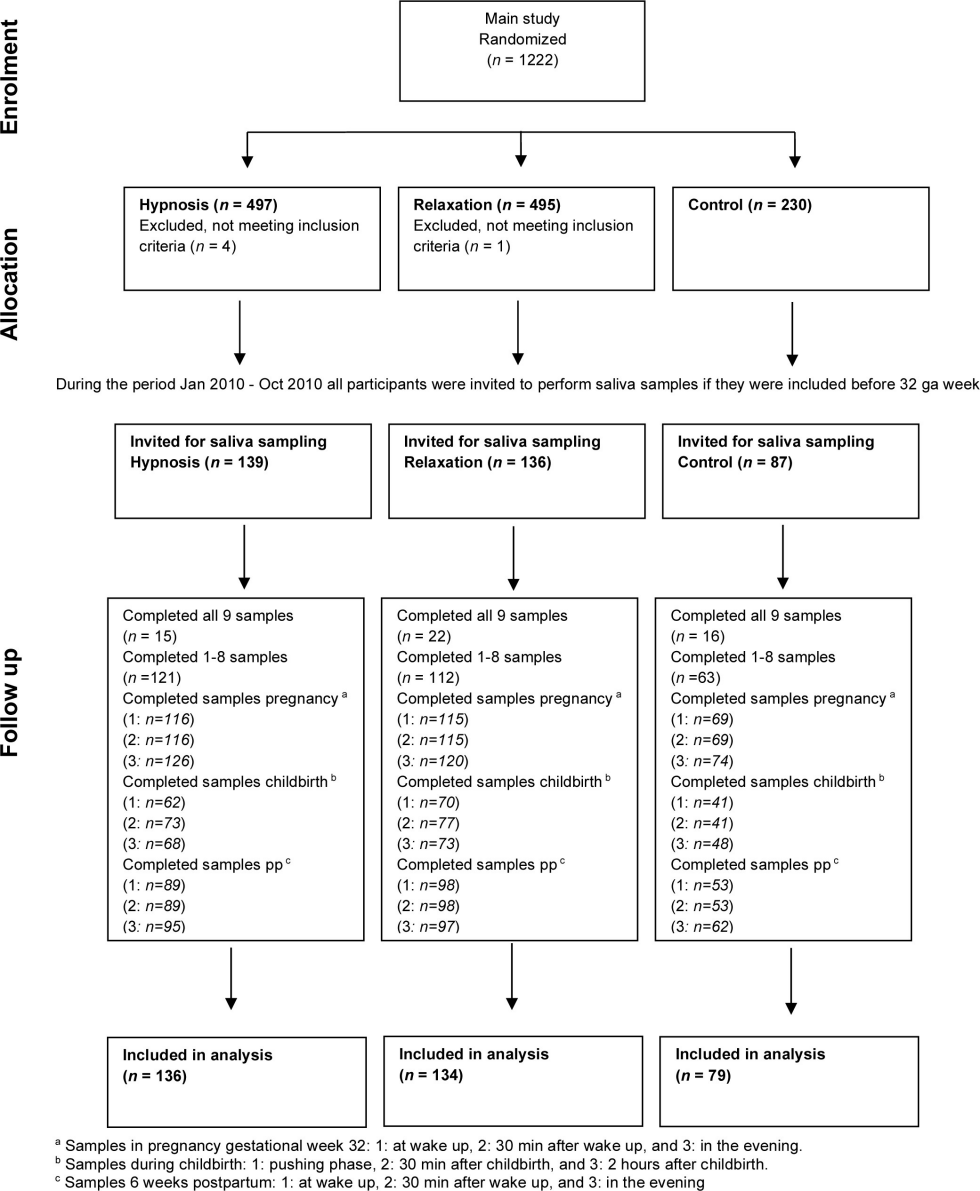
Flow diagram of enrolment, allocation, and study participation.

In the main study, 1222 women were randomized. We excluded 5 of these participants since they did not meet the inclusion criteria. In total, 362 women were enrolled in the study before their 32nd gestational week and invited to collect salivary cortisol samples. Of these, 349 (96.4%) accepted. Of the 349 participants, 84.8% collected at least one sample, and 15.2% collected all 9 samples. During pregnancy at gestational week 32, the number of collected saliva cortisol samples were 300 at awakening, 300 thirty minutes after awakening, and 320 in the evening. During childbirth, we collected 173 samples in the beginning of the pushing phase, 198 samples thirty minutes after the childbirth, and 189 samples 2 hours after childbirth. Six weeks after childbirth, the number of collected saliva cortisol samples were 240 at awakening, 240 thirty minutes after awakening, and 254 in the evening. (See Fig 1, Flowchart).

### Characteristics of the participants

Baseline and obstetric childbirth information were available for all participants. The questionnaire providing information about childbirth experience was answered by 98.9% of the participants. The three groups were generally comparable, including wellbeing in pregnancy, experienced calmness during labor and childbirth, and experienced pain intensity. However, use of epidural analgesia differed between the three groups as the usual care group had a much lower frequency than the two other groups (20.3% compared to approximately 33%). According to mode of delivery, more women in the hypnosis group (16.9%) gave birth by emergency cesarean section compared to the relaxation group (13.4%) and the usual care group (10.3%). Length of birth from admission to the birth department until childbirth was more than 1 hour shorter in the usual care group (5.97 hour) compared to the relaxation group (7.41 hour) and the hypnosis group (7.01) (Table 1).

**Table 1:** Participant characteristics.

	Hypnosis (n = 136)	Relaxation (n = 134)	Care as usual (n = 79)
Age (years), mean (SD)	29.8(3.4)	30.0(3.3)	29.8(3.5)
Pre-pregnant BMI (kg/m2), mean (SD)	22.3(2.7)	22.7(3.3)	22.6(3.4)
Smoking, during pregnancy, n (%)	1(0.7)	1(0.7)	3(3.8)
Higher education (years beyond high school), (n) %			
None	3(2.2)	0(0)	1(1.3)
1–4 years	72(52.9)	74(55.2)	41(51.9)
4 years and longer	61(44.9)	60(44.7)	37(46.8)
Living with partner, (n (%)	133(97.8)	133(99.3)	78(98.7)
Partner present at childbirth, n (%)	130(97.7)	133(99.3)	77(98.2)
WHO-5 well-being index, (max score 100), mean (SD)	70.6(13.3)	69.2(13.1)	67.9(13.7)
Compliance to intervention ^**a**^, n (%)	136(100)	131(97.8)	
Additional antenatal training^**b**^, n (%)	76(55.9)	71(53.0)	45(57)
Mode of delivery			
Spontaneous, n (%)	95(69.9)	93(69.4)	60(75.9)
Assisted, n (%)	15(11.0)	17(12.7)	10(12.7)
Cesarean section, emergency, n (%)	23(16.9)	18(13.4)	1(10.1)
Cesarean section, scheduled, n (%)	3(2.2)	6(4.5)	6(1.3)
Perceived calmness ^**c**^, (max score 10), mean (SD)	7.7(2.2)	7.2(2.6)	7.3(2.6)
Experienced pain ^**d**^, (max score 10), mean (SD)	6.7(2.9)	7.1(2.5)	7.2(2.5)
Experienced pain ^e^, (max score 10), mean (SD)	6.9(2.5)	7.0(2.5)	7.1(2.7)
Length of birth			
Pushing phase ^**f**^, hours, mean (SD)	0.75(0.55)	0.77(0.44)	0.61(0.36)
Admission to birth ^**g**^, hours, mean (SD)	7.02((4.69)	7.41(4.96)	5.97(4.07)
Use of epidural analgesia, n (%)	46(33.8)	44(32.8)	16(20.3)
Breastfeeding 6 weeks postpartum, n (%)	125(94.0)	123(91.8)	73(93.6)

^**a**^ Attending at least one of three classes

^**b**^Attending additional antenatal training offered by private providers

^**c**^Perceived calmness during labor and childbirth, range 0–10, max score 10

^**d**^Pain intensity during labor at the end of the active phase, range 0–10, max score 10

^**e**^Pain intensity during the pushing phase, range 0–10, max score 10

^**f**^ Length of pushing phase

^**g**^Time from admission to the birth department until childbirth

### Cortisol during pregnancy and at 6 weeks postpartum

The mean cortisol levels, CAR, and CDD for each group at gestational week 32 and 6 weeks postpartum are shown in Table 2. For all three groups, cortisol showed a peak at wake up and a decrease over the day in pregnancy and 6 weeks postpartum.

**Table 2:** Saliva cortisol concentration over the day according to randomization group and between group differences in pregnancy and 6 weeks postpartum.

Sampling time	Hypnosis *(n)* Mean(SD) nmol/l	Relaxation *(n)* Mean(SD) nmol/l	Care as usual *(n)* Mean(SD) nmol/l	Relaxation/Hypnosis Ratio cortisol (CI) p	Usual care /Hypnosis Ratio cortisol (CI) p
**Samples Pregnancy ga** ^**b**^ **32**					
Wake up	*(n = 116)* 16.8 (6.0)	*(n = 115)* 16.6(5.8)	*(n = 69*) 15.0(5.6)	0.99 (0.90;1.09)0.91	0,88 (0.78;0.99)0.02
30 min after wake up	*(n = 116)* 22.0(6.8)	*(n = 115)* 22.7(9.9)	*(n = 69)* 22.5(11.0)	0.97 (0.88;1.06)0.53	1.07 (0.95;1.22)0.27
Evening 20 a clock	*(n = 126)* 4.6(1.9)	*(n = 120)* 5.0(2.3)	*(n = 74)* 4.9(1.9)	1.08 (0.99;1.19)0.09	1.20 (1.04;1.37)0.01
	*(n)* Mean(CI)	*(n)* Mean(CI)	*(n)* Mean(CI)		
CAR* per 30 min	*(n = 115)* 4.1(3.4;4.9)	*(n = 115)* 3.2(2.5;4.0)	*(n = 69)* 4.1(3.3;5.3)		
CDD** per 1 hour	*(n = 109)* 1.5(1.4;1.6)	*(n = 114)* 1.4(1.2;1.5)	*(n = 66)* 1.3(1.2;1.5)		
**Samples 6 weeks postpartum**					
Wake up	*(n = 89)* 10.3(6.3)	*(n = 98)* 10.0(67)	*(n = 53)* 9.2(3.5)	1.06 (0.87;1.27)0.58	0,98 (0.78;1.22)0.85
30 min after wake up	*(n = 89)* 15.6(12.7)	*(n = 98)* 15.4(17.5)	*(n = 53)* 12.9(5.1)	1.07 (0.88;1.29)0.51	0.99 (0.77;1.27)0.93
Evening 20 a’ clock	*(n = 95)* 2.3(3.6)	*(n = 97)* 2.4(4.5)	*(n = 62)* 2.9(10.7)	1.04 (0.86;1.25)0.68	1.01 (0.77;1.32)0.96
	*(n)* Mean(CI)	*(n)* Mean(CI)	*(n)* Mean(CI)		
CAR ^**c**^, nmol/l per 30 min	*(n = 88)* 2.4(1.9;3.1)	*(n = 98)* 2.4(1.9;3.0)	*(n = 52)* 1.9(1.4;2.6)		
CDD ^d^ nmol/l per 1 hour	*(n = 80)* 1.0(0.9;1.1)	*(n = 87)* 1.0(0.9;1.1)	*(n = 49)* 0.9(0.8;1.1)		

^a^ Ratio of saliva cortisol concentrations

^b^Gestational week

^c^CAR: Cortisol awakening response

^d^CDD: Cortisol decline over the day

Between group differences are presented in Table 2. For pre-intervention data obtained during pregnancy, the cortisol concentration was found to be 12% lower in the usual care group than in the hypnosis group at wake up (0.88 (95% CI: 0.78–0.99)). Thirty minutes after wake up, the concentration tended to be higher in the usual care group (1.07 (95% CI: 0.95–1.22)), and in the evening the concentration was 20% higher in the usual care group (1.20 (95% CI: 1.04–1.37)). Between the relaxation group and the hypnosis group, we found no statistical differences at the three time points. These results did not change when adjusting for the prespecified potential confounders.

Six weeks postpartum, no statistical differences were found at any time point between the usual care group and the hypnosis group or the relaxation group and the hypnosis group, respectively (Table 2). Adjusting for the prespecified potential confounders did not change these results.

#### Association between cortisol and well-being in pregnancy

For the total sample, we found no associations between wellbeing at baseline and wake up cortisol concentration (rs -0.08, p:0.15), between wellbeing at baseline and CAR (rs -0.09, p:0.14) in pregnancy, or between wellbeing at baseline and CDD in pregnancy (rs -0.04, p:0.55).

### Cortisol during childbirth

The mean cortisol level for each group during childbirth and the differences across the three groups are shown in Table 3. For all three groups, the highest cortisol level was observed 30 minutes after childbirth and the lowest 2 hours after childbirth.

**Table 3:** Saliva cortisol concentration according to randomization group and between group differences during childbirth.

Sampling time	Hypnosis *(n)* Mean(SD) nmol/l	Relaxation *(n)* Mean(SD) nmol/l	Care as usual *(n)* Mean(SD) nmol/l	Relaxation/Hypnosis Ratio cortisol^a^ (CI) p	Usual care /Hypnosis Ratio cortisol^a^ (CI) p
**All participants**					
Beginning pushing phase	*(n = 62)* 154.8 (11.9)	*(n = 70)* 110.1(88.6)	*(n = 41*) 126.7(79.4)	0.79 (0.59;1.04)0.10	0,83 (0.61;1.15)0.26
30 min after childbirth	*(n = 73)* 209.0(157.8)	*(n = 77)* 207.7(138.1)	*(n = 48)* 189.5(111.8)	0.96 (0.73;1.25)0.75	0.82 (0.58;1.13)0.22
2 hours after birth	*(n = 68)* 90.6(69.4)	*(n = 73)* 79.3(58.1)	*(n = 48)* 77.7(47.0)	0.79 (0.60;1.04)0.09	0.75 (0.54;1.06)0.11
**Vaginal and emergency cesarean section birth**					
Beginning pushing phase	*(n = 61)* 154.4(111.9)	*(n = 65)* 111.4(88.5)	*(n = 38)* 126.7(79.4)	0.81 (0.61;1.06) 0.13	0,86 (0.62;1.18) 0.35
30 min after childbirth	*(n = 72)* 209.0(157.8)	*(n = 74)* 210.1(137.4)	*(n = 46)* 196.2(109.3)	0.99 (0.76;1.29) 0.93	0.84 (0.61;1.16) 0.30
Evening 20 a’ clock	*(n = 64)* 90.6 (69.4)	*(n = 70)* 80.2(58.0)	*(n = 46)* 77.7(47.0)	0.80 (0.61;1.06) 0.12	0.77 (0.55;1.09) 0.14
**Vaginal and emergency cesarean section birth**				***Adjusted ratio (CI)***^***b***^***p***	***Adjusted ratio (CI)***^***b***^***p***
Beginning pushing phase	*(n = 61)* 154.4(111.9)	*(n = 65)* 111.4(88.5)	*(n = 38)* 126.7(79.4)	0.73 (0.56;0.95) 0.02	0,77 (0.57;1.03) 0.08
30 min after childbirth	*(n = 72)* 209.0(157.8)	*(n = 74)* 210.1(137.4)	*(n = 46)* 196.2(109.3)	0.92 (0.72;1.17) 0.48	0.77 (0.57;1.05) 0.10
2 hours after childbirth	*(n = 64)* 90.6 (69.4)	*(n = 70)* 80.2(58.0)	*(n = 46)* 77.7(47.0)	0.76 (0.59;0.97) 0.03	0.72 (0.52;0.99) 0.05

^a^Ratio of saliva cortisol concentrations

^b^Ratio of saliva cortisol concentrations adjusted for use of epidural analgesia and mode of delivery

^cb^The weighted adjusted p-values by Holm’s-Bonferroni’s method: p-value1 < 0.0083, p-value2 < 0.0010, p-value3 < 0.0125 p-value4 < 0.0167, p-value5 < 0.0250, p-value6 < 0.0500

In the intention to treat analysis, we found no differences in cortisol levels across the three groups at the beginning of the pushing phase. Excluding women giving birth by planned cesarean section from the analysis did not change these results. When we added mode of delivery and use of epidural to the model, we observed a 27% lower cortisol concentration in the relaxation group compared to the hypnosis group (0.73 (95% CI: 0.56–0.95)). When comparing the usual care group with the hypnosis group, the difference was border significant (0.77 (95% CI: 0.57–1.03)).

Further adjustment for length of birth, season of birth, additional antenatal training offered by private providers, BMI, mothers age, smoking, well-being at baseline and education did not change these results.

The intention to treat analysis 30 minutes after childbirth revealed no differences when comparing the relaxation group with the hypnosis group (0.96 (95%, CI: 0.73–1.25), but the usual care group had 18% lower cortisol levels than the hypnosis group (0.82 (95% CI: 0.58–1.13)). The results did not change significantly by excluding participants giving birth by elective cesarean section from the analysis or by adding mode of delivery and use of epidural analgesia to the model. Adjusting for further potential confounders mentioned above did not change these results.

Two hours after childbirth, the intention to treat analysis revealed no differences across the three groups. Excluding women giving birth by planned cesarean section from the analysis did not change the results. When adding mode of delivery and use of epidural analgesia to the model, we found a 24% lower cortisol concentration in the relaxation group compared to the hypnosis group (0.76 (95% CI: 0.59–0.97)). In the usual care group, concentrations were 28% lower compared to the hypnosis group (0.72 (95% CI: 0.52–0.99)). Further adjustment for potential confounders mentioned above did not change these results.

In the sensitivity analysis, 49 participants were excluded due to missing cortisol samples at wake up and 30 minutes after wake up in the pregnancy. The sensitivity analyses after multiple imputation of missing cortisol values did change any of the results significantly (S1 Table).

#### Correlation between cortisol and experienced pain and calmness

In the total sample, we examined correlations between cortisol concentration and experienced pain and calmness according to mode of delivery and use of epidural analgesia. We only observed a correlation between experienced pain in the end of the pushing phase and higher cortisol concentration 30 minutes after childbirth for women giving instrumental birth without epidural analgesia (0.56 (p = 0.04)) (Table 4).

**Table 4:** Correlation between cortisol concentration and length of birth, experienced pain and calmness during childbirth.

	Cortisol concentration		
	Beginning pushing phase rs ^a^ (p value), *n*	30 min after childbirth rs ^a^ (p value), *n*	2 hours after childbirth rs ^a^ (p value), *n*
**All participants**			
Perceived calmness ^**b**^	0.10 (0.24), *142*	0.06 (0.43), *195*	0.02 (0.78), *149*
Experienced pain active phase ^**c**^	-0.02 (0.82), *139*		
Experienced pain pushing phase ^**d**^	0.09 (0.30), *139*	0.13 (0.07), *188*	
Birth length			
Admission to pushing phase ^**e**^	-0.02 (0.80), *138*		
Pushing phase ^**f**^		0.16(0.03), *187*	
Admission to birth ^**g**^		-0.10(.18), *198*	-0.04(0.61), *150*
**All spontaneous birth**			
Perceived calmness ^**b**^	0.09 (0.28), *136*	0.05 (0.54), *186*	0.05 (0.58), *143*
Experienced pain active phase ^**c**^	-0.02 (0.80), *136*		
Experienced pain pushing phase ^**d**^	0.09 (0.27), *136*	0.12 (0.11), *186*	
Birth length			
Admission to pushing phase ^**e**^	-0.01(0.89), 117		
Pushing phase ^**f**^		0.18(0.02), 160	
Admission to birth ^**g**^		-0.06(0.44), 160	-0.04(0.69),127
**Spontaneous birth without epidural analgesia**			
Perceived calmness ^**b**^	0.14 (0.15), *99*	0.01 (0.90), *136*	0.12 (0.21), *107*
Experienced pain active phase ^**c**^	-0.12 (0.25), *99*		
Experienced pain pushing phase ^**d**^	0.00 (0.98). 99	0.03 (0.71), *136*	
Birth length			
Admission to pushing phase ^**e**^	0.02(0.85), 93		
Pushing phase ^**f**^		0.14(0.13), 124	
Admission to birth ^**g**^		0.26(0.01), 124	-0.13(0.21),99
**Spontaneous birth with epidural analgesia**			
Perceived calmness*	-0.04 (0.81), *37*	-0.11 (0.40), *50*	-0.13 (0.47), *36*
Experienced pain active phase ^**c**^	0.02 (0.91), *37*		
Experienced pain pushing phase ^**d**^	0.29 (0.07), *37*	0.22 (0.13), *50*	
Birth length			
Admission to pushing phase	0.32(0.13), 24		
Pushing phase ^**f**^		0.21(0.21), 36	
Admission to birth^**g**^		0.01(0.97), 36	0.02(0.93),28
**All instrumental birth** ^h^			
Perceived calmness ^**b**^	0.13 (0.54), *25*	0.03 (0.89), *35*	-0.27 (0.22), *22*
Experienced pain active phase ^**c**^	-0.06 (0.79), *22*		
Experienced pain pushing phase ^**d**^	-0.20 (0.38), *22*	0.32 (0.11), *26*	
Birth length			
Admission to pushing phase ^**e**^	-0.16(0.49), 21		
Pushing phase ^**f**^		0.03(0.89), 27	
Admission to birth ^**g**^		-0.07(0.69), 36	-0.25(0.27),22
**Instrumental birth** ^h^ **without epidural analgesia**			
Perceived calmness ^**b**^	-0.13 (0.54), *7*	-0.41 (0.11), *16*	-0.05 (0.89), *10*
Experienced pain active phase ^**c**^	-0.04 (0.94), *7*		
Experienced pain pushing phase ^**d**^	-0.19 (0.69), *7*	0.56 (0.04), *14*	
Birth length			
Admission to pushing phase	0.54(0.27), 6		
Pushing phase ^**f**^		0.33(0.27), 3	
Admission to birth ^**g**^		0.24(0.36), 17	-0.61(0.06),10
**Instrumental birth** ^h^**with epidural analgesia**			
Perceived calmness ^**b**^	0.28 (0.26), *18*	0.30 (0.21), *19*	-0.40 (0.20), 12
Experienced pain active phase ^**c**^	0.05 (0.85), *15*		
Experienced pain pushing phase ^**d**^	0.37 (0.16), *15*	0.21 (0.47), *14*	
Birth length			
Admission to pushing phase	-0.45(0.09), 15		
Pushing phase ^**f**^		-0.07(0.81), 14	
Admission to birth ^**g**^		0.23(0.34), 19	-0.08(0.78),12

^**a**^Spearman's coefficient

^**b**^Perceived calmness during labor and childbirth, range 0–10, max score 10

^**c**^Experienced pain intensity during labor at the end of the active phase, range 0–10, max score 10

^**d**^ Experienced pain intensity during the pushing phase, range 0–10, max score 10

^**e**^ Time from admission to the birth department until pushing phase

^**f**^ Length of pushing phase

^**g**^ Time from admission to the birth department until childbirth

^**h**^ Assisted and emergency cesarean birth

When we explored the correlation between the duration of birth and cortisol, we observed a correlation between the length of the pushing phase and the cortisol concentration 30 minutes after childbirth for all participants (0.16 (p = 0.03)) and for women with spontaneous birth (0.18 (p = 0.02)). A correlation was also seen for the length from arriving to the birth department until childbirth for women giving spontaneous birth without epidural analgesia (0.31(p = 0.00)).

## Discussion

In this study, we aimed to examine the effect of a brief antenatal training course in self-hypnosis on the cortisol response during childbirth and 6 weeks postpartum. As comparison groups, we included a brief antenatal training course in relaxation and usual care. During childbirth, we found, contrary to our hypothesis, a general tendency towards higher cortisol concentrations in the hypnosis group compared to the other two groups in the beginning of the pushing phase and 2 hours after childbirth. We found no differences at the other timepoints around childbirth. Neither did we observe any differences in daily cortisol response across the three groups 6 weeks postpartum.

In pregnancy, the women collected the cortisol samples in gestational week 32 before the interventions took place. For each group, we observed, as expected, a characteristic diurnal rhythm in cortisol showing a peak at wake up and a decrease over the day [8, 10, 28]. The cortisol concentrations differed between the hypnosis group and the usual care group at wake up and in the evening. Between the hypnosis group and the relaxation group, no differences were found at any time point in pregnancy. As cortisol in some studies has been suggested to be related to psychological wellbeing [28–30], we tested the correlation between the cortisol response and wellbeing in pregnancy and found no correlation. This corresponds to other studies failing to show a correlation between wellbeing and cortisol in pregnancy [31–33]. Between group differences in cortisol levels could also be explained by individual variations [5, 46]. Consequently, we included the wake up cortisol concentration and the CAR from pregnancy in our statistical model.

Six weeks after childbirth, no differences in cortisol response across the study groups were seen. As expected, we observed a diurnal rhythm in cortisol. The mean concentrations were significant lower 6 weeks postpartum compared to the concentrations during pregnancy. These findings are in line with previous studies [8–10, 15].

The concentrations of cortisol were higher during childbirth compared to the concentrations during pregnancy and 6 weeks after childbirth. This corresponds with current knowledge [5, 10]. The cortisol level during labor and childbirth are reflecting an extremely demanding experience with high psychophysiological stress. One of the effects of cortisol is to help the body to adapt to stress and gain energy by influencing metabolic activities [47]. For all three groups, the cortisol concentration peaked 30 minutes after childbirth, and they were all able to turn off the allostatic response 2 hours after childbirth.

We did not observe any differences in cortisol concentrations in the hypnosis group compared to the other two groups at the measured time points around the childbirth in the intention to treat analysis. Use of epidural analgesia and mode of delivery are well known factors interacting with the cortisol concentrations during childbirth [12, 13, 16–18, 48] and these factors were not evenly distributed across the three groups. We controlled for this by excluding women with a planned cesarean birth and including use of epidural analgesia and mode of delivery in our statistical model. This led to higher cortisol concentrations in the hypnosis group compared to the other two groups in the beginning of the pushing phase and 2 hours after childbirth.

Higher concentrations of cortisol could be interpreted as an indicator of higher levels of pain, emotional stress and fear during labor and childbirth [12, 14]. Therefore, we examined the correlations between the cortisol concentrations and the women’s experienced calmness and pain during labor and childbirth. However, we only observed correlations between experienced pain in the pushing phase and cortisol concentration 30 minutes after childbirth in women giving instrumental birth without epidural analgesia.

When we explored the correlation between the cortisol concentrations and the length of birth, we only observed rather weak correlations for the concentrations 30 minutes after childbirth for all women, for women giving spontaneous birth, and for women giving spontaneous birth without epidural analgesia. When we adjusted for length of birth in the comparison of cortisol levels across treatment groups, the results did not change even though length of birth differed across the groups.

We do not know how to explain the differences between the hypnosis group and the other two groups. It may be that self-hypnosis, contrary to expectations, increase stress during childbirth. The increase in cortisol could be due to some women experiencing hypnosis as a challenging task provoking an emotional stress response, but we had no data to explore this. The differences in cortisol concentration at the time points around childbirth may also be interpreted as a well-functioning allostatic response [5]. Thus, the hypnosis group was perhaps more able to mobilize the appropriate energy to the body during labor and 2 hours after childbirth [7, 47]. However, these explanations are only guesses which cannot be confirmed by our data.

To our knowledge, no previous studies have investigated the effect of antenatal self-hypnosis on cortisol. Hypnosis interventions including guided imagery and relaxation have for example been investigated in HIV-seropositive women [49] and in students [50–52] with cortisol as an outcome. Results suggest that these interventions may be partially responsible for decreases in cortisol. Childbirth is a very different situation, though, and our study may not be comparable with these studies.

Benfield et al have emphasized the complexity by using cortisol as a stress marker in term labor and recommend to pay attention to methodological issues [5]. The same remains when using cortisol as a biomarker for wellbeing in the postpartum period [6]. We did consider the methodological issues and used an RCT set up. We aimed for a homogeneous population by only including nulliparous healthy women with a BMI lower than 30. The participants were asked to carefully follow the instruction when collecting the saliva samples. We took the baseline cortisol into account, performed the analysis as intention to treat, and we took prespecified confounders into account.

We had noncompliance on the sampling of saliva and that may limit our results. The majority of the invited participants provided us with at least one sample but we only succeeded collecting all 9 samples from 15% of the invited participants. As most samples were missing during childbirth, we performed sensitivity analysis for these timepoints. These analyses did not suggest that the observed results were affected by the missing values. However, the post hoc tests do not confirm the results. Also, we do not know whether the lack of findings is simply due to a power issue. Therefore, our results should be interpreted with caution.

In conclusion, our results suggest that a brief course in antenatal hypnosis training compared to a short course in relaxation and usual care did not have any influence on saliva cortisol concentration 6 weeks postpartum. The intervention may influence the release of cortisol during the extreme labor work and the first hours after childbirth with no long-term consequences. However, further research is needed to confirm and help interpret these findings.

## Supporting information

S1 Checklist CONSORT 2010 checklist of information to include when reporting a randomised trial*. (DOCX)

S1 Table Sensitivity analyses of saliva cortisol concentration according to randomization group and between group differences during childbirth. (DOCX)

S1 File (DOC)
