# Supplementary material for: Effects of antenatal hypnosis on maternal salivary cortisol during childbirth and six weeks postpartum—A randomized controlled trial
Source: PLoS One. 2020 May 1;15(5):e0230704. doi: 10.1371/journal.pone.0230704 (PMC7194394; doi:10.1371/journal.pone.0230704)
Supplement: S1 Table — (DOCX) [file pone.0230704.s002.docx]

**S1 Table: Sensitivity analyses of saliva cortisol concentration according to**

**randomization group and between group differences during childbirth**

| **Sampling time** | **Relaxation/Hypnosis**  Crude ratio (CI)  p | **Usual care /Hypnosis**  Crude ratio (CI)  p |
| --- | --- | --- |
| **All participants** |  |  |
| *Beginning pushing phase* | *0.80 (0.61;1.05)*  *0.34* | *0,89 (0.65;1.21)*  *0.14* |
| *30 min after childbirth* | *0.95 (0.73;1.23)*  *0.69* | *0.82 (0.60;1.11)*  *0.20* |
| *2 hours after birth* | *0.78 (0.60;1.02)*  *0.07* | *0.76 (0.55;1.05)*  *0.10* |
| **Participants vaginal/**  **Instrumental birth** |  |  |
| *Beginning pushing phase* | *0.82 (0.63;1.07)*  *0.15* | *0,92 (0.68;1.25)*  *0.60* |
| *30 min after childbirth* | *0.98 (0.75;1.26)*  *0.86* | *0.84 (0.62;1.14)*  *0.28* |
| *2 hours after birth* | *0.79 (0.61;1.03)*  *0.08* | *0.78 (0.56;1.07)*  *0.13* |
| **Participants vaginal/**  **Instrumental birth** | ***Adjusted ratio (CI)^b^***  ***p*** | ***Adjusted ratio (CI)^b^***  ***p*** |
| *Beginning pushing phase* | *0.76 (0.62;0.97)*  *0.03* | *0,82 (0.61;1.10)*  *0.18* |
| *30 min after childbirth* | *0.91 (0.72;1.16)*  *0.47* | *0.79 (0.58;1.06)*  *0.11* |
| *2 hours after birth* | *0.75 (0.59;0.96)*  *0.02* | *0.73 (0.54;1.00)*  *0.05* |

^a^ Missing values predicted by randomization group, mode of delivery, use of epidural analgesia, length of birth, and BMI

^b^Adjusted for use of epidural analgesia and mode of delivery
