# Supplementary material for: Effects of antenatal hypnosis on maternal salivary cortisol during childbirth and six weeks postpartum—A randomized controlled trial
Source: PLoS One. 2020 May 1;15(5):e0230704. doi: 10.1371/journal.pone.0230704 (PMC7194394; doi:10.1371/journal.pone.0230704)
Supplement: S1 File — (DOC) [file pone.0230704.s003.doc]

**Protecol 2008**

**Mental training and childbirth: the effect on pain experience, duration of labour and other birth outcomes**

**Introduction**

**Childbirth and pain**

Labour pain, which represents a great challenge for birthing women, can lead to a negative childbirth experience. Together with loss of control, labour pain accounts for one of the most important factors for a negative childbirth experience [1;2]. A traumatic childbirth experience may result in postpartum depression, posttraumatic stress syndrome, future caesarean section, or a reluctance to have more children [3–5].

Pharmacological methods to relieve labour pain are limited because almost all drugs pass the placental barrier and thereby may have a negative impact on the fetus [6]. Today, epidural analgesia is considered the most effective pain relief method. This technique is, however, associated with side effects such as instrumental deliveries as well as more serious adverse effects for the parturient woman [7]. Furthermore, epidural and other medical analgesia to relieve labour pain may disturb the newborns’ temperature regulation and breastfeeding and crying behaviours [8].

In Denmark, almost 64,000 children are born every year [9]. It is a very important task for society to endeavour to support the parturient woman in an appropriate way to prevent a negative childbirth experience. By focusing on prevention and health promotion, personal and economical costs can be minimised.

**Childbirth, pain, and stress hormones**

Childbirth is an stressful experience, and the concentration of stress hormones is generally higher during childbirth than during pregnancy [10-13]. Pain relief reduces the level of stress hormones, and there is evidence that the levels of both anxiety and pain correlate with the concentration of stress hormones during childbirth [11;14]. Cortisol is a common biomarker for the stress response [15;16].

The measured levels of stress hormones in umbilical cord blood are an indicator of the child’s condition. It has been suggested that stress during pregnancy, childbirth, and the first part of life may affect the programming of the HPA axis in children, with possible consequences for the child's stress sensitivity, behaviour, and attention span later in life [11;17-19].

**Mental techniques for pair relief**

“Mind Body Medicine” is an overall term for various interventions based on the interaction between mind and body. In the industrialised world, there is an increasing interest in these techniques among the adult population. Hypnosis and relaxation are considered internationally to belong to the field of “Mind Body Medicine”[[1]](#footnote-2) [20]. Relaxation can have a beneficial effect in the treatment of depressive conditions and when used by pregnant women to handle the anxiety and stress of giving birth [21;22]. Hypnosis has long been used in connection with various health-related conditions, and it has been shown to have a positive effect in the treatment of several common illnesses [20].

Pain has been one of the indications for this treatment because experience and research show that through use of this technique there is a markedly increased responsiveness of a series of basic physiologic processes and systems, such as pain, the circulation, immunity and muscular and autonomic nerve activity [23]. In studies in which functional magnetic resonance scanning was used, it has been shown that pain hypnosis can change cortical activity in areas of the brain that are important for the generation of the pain [24;25]. Hypnosis has been evaluated as a technique to relieve pain in several experimental studies. Hypnosis has been shown to have a positive effect on both internal pain and external pain, e.g. phantom pain, muscular pain, neurogenic pain and pain due to burns [26;27].

**Studies of childbirth and hypnosis**

Systematic reviews conclude that hypnosis can be advantageous for the birthing woman. Thus the use of pharmacological pain relief and medical interventions can be reduced through this technique [28-30]. A metaanalysis of randomised controlled studies of the use of hypnosis in birthing women found a reduction (relative risk (RR): 0.51; 95% confidence intervals (CI) 0.28–0.95) in the use of pharmacological pain relief. The use of oxytocin augmentation was also reduced (RR: 0.31; 95% CIs: 0.18–0.52).

Studies have shown other effects in pregnant women trained in the use of self-hypnosis. The results indicate that it is possible to achieve a higher pain threshold, more spontaneous births, higher birth weights, improve Apgar scores, prevent preterm delivery and reduce the duration of labour, fear and stress during birth, and postpartum depressions [24;31-35]. Hypnosis may also be applicable to a broad group of women, including target groups that may otherwise be difficult to reach such as vulnerable women [34;36] (table 1, appendix 1).

Generally, there are problems concerning the size of the study groups and the quality of the methods used in many of the studies in this area. For example, the case-control study design is associated with a great risk of false interpretation, most of the randomised studies are small, some studies are not even randomised, and the methods used are not well described. In a metaanalyis of the use of hypnosis during childbirth conducted by Cyna et al. in 2004, only four randomised studies could be included due to methodologic determinants [35-38]. In a systematic literature search in PubMed, the Cochrane Library, and Cinahl, we were able to identify only one randomised controlled study of good methodologic quality [34].

Considering that hypnosis has been reported to have an exceptionally good effect, one can wonder why hypnosis is not more widely used in the healthcare system. This mental training technique has, however, been associated with misunderstandings and negative associations [39]. Unfortunately, as mentioned, the many methodologic problems in many of the studies performed in this area have resulted in a great risk of misinterpretation of the results of these studies. There is therefore need of a well-performed randomised study that includes a sufficient number of birthing women [28-30]. Only by performing such a study can it be finally determined whether self-hypnosis has so many advantages that it should be offered to pregnant women.

For this reason, a large, randomised, controlled study was begin in Australia in 2006 with the primary objective of evaluating the effect of hypnosis on use of medical pain relief [40]. The research team reported a positive effect of a mental training programme developed at their centre and in use for more than 10 years. The intervention involved three 1-hr periods of instruction in self-hypnosis, with a follow-up CD after each visit [24]. Self-hypnosis as a mental training technique has not been evaluated in a Danish obstetric context, and this is important because the organisation and procedures in the Danish healthcare service are different from those in other countries just as is the cultural and mental background of Danish women. If three 1-hr instruction sessions with a follow-up CD can have an effect on labour pain, a big problem could be alleviated with the use of few resources.

It is also of great societal importance to determine whether this mental pain relief technique could influence the choice of future modes of birth and thereby break the rising curve of caesarean deliveries. In addition, it is of great importance to investigate techniques that might reduce the large number of epidural blockages given during childbirth and to study new, effective techniques that can relieve labour pains without side effects. For these reasons, the present project was initiated in dialogue with the Australian research group.

**Aim**

The aim of the study is to investigate the effect of self-hypnosis during childbirth on the following endpoints:

Primary endpoint:

- The use of epidural analgesia during labour

Secondary endpoints:

- Duration of labour
- Birth progression at arrival at birth department
- Birth experience (pain, control, anxiety)
- Medical interventions during birth including mode of delivery
- Saliva cortisol profile at birth and 6 weeks post partum (p.p.)
- Infection during labour and the first 6 weeks p.p. (mother and child)
- Postnatal depression
- Breastfeeding duration
- Child's condition and wellbeing at birth and 6 months later
- Future mode of delivery

**Methods**

The project will be performed as two studies, a pilot study and a main study.

Design and material

The project will take place at the Obstetrics Department at Aarhus University Hospital, Skejby. The main study will be designed as a randomised 3-arm controlled trial consisting of

- *An intervention group* will attend three classes on self-hypnosis for childbirth in the last trimester of the pregnancy. The programme will include three audio recordings for home use as well as an audio recording especially meant during labour. The training programme and the audio recordings will be based on the experiences from a Australian project [40] and in corporation with a group of consultants.
- *An active comparison group* will attend three antenatal classes on a variety of body awareness, relaxation, and mindfulness techniques. This course will also include audio recordings for home use and during labour.
- *The control group* will receive ordinary antenatal care, which includes a nuchal translucency scan at about gestational week 12, an anomaly scan at about gestational week 19, four to five visits to the midwifery clinics, and a tour of the birth department.

Blinding

The women in the invention group and in the active comparator group will be informed about what kind of training they have been randomised to receive. We aim to blind the staff at the Obstetric Department. They will be informed only that the research project will investigate two different kinds of mind-body, self-hypnosis, and relaxation techniques, as two equally effective approaches. The data manager will also be blinded with regard to the participants’ allocated treatment.

Inclusion:

All healthy nulliparous women (women with gestational diabetes will be included, BMI < 39 will be excluded) fulfilling the criteria: older than 18 years, uncomplicated pregnancy, able to understand and speak Danish, and planning to give birth at Aarhus University Hospital, Skejby.

All the women showing an interest in participating will be invited to an information meeting.

Data and data sources:

The data collection will include questionnaires, register data from “The Aarhus Birth Cohort”, medical records, and biological material. For further information, please see table 1

**Table 1: Flow and data sources**

| **When** | **What/where** | **Data source** |
| --- | --- | --- |
| Gestational week 10 |  | * Questionnaire from “The Aarhus Birth Cohort” |
| Gestational week 29 | Written information |  |
| Gestational weeks 31–33 | Information meeting |  |
| Gestational weeks 32–34 | Enrolment in the project | * Questionnaire 1 including Wijma Delivery Expectancy/ Experience Questionnaire 1 (*W-DEQ*) |
| Gestational weeks 34–36 | Start of intervention | *Test for hypnotic susceptibility in the intervention group |
| Gestational week 35 |  | *Saliva cortisol sample x 3 |
| Labour | Labour | * Saliva cortisol sample x 3  * Blood sample cortisol umbilical cord (only if economically possible)  *“The Aarhus Birth Cohort  *Medical records |
| 6 weeks p.p.^2^ |  | * Saliva cortisol sample x 3  * Questionnaire 1 including Wijma Delivery Expectancy/ Experience Questionnaire 1 (*W-DEQ*) and Edinburgh Postnatal depression scale  * Medical records |
| 6 month. p.p. |  | * Questionnaire 3 including Edinburgh Postnatal depression scale |

2. p.p.= post partum (after childbirth)

Saliva cortisol will function as an indicator of the endocrine stress response. In this part of the project, we will only include 260 participants. Thus 100 participants from the intervention group, 100 participants from the active comparison group, and 100 participants from the control group will be included.

**Pilot study:**

Before starting the main study, a pilot study will be performed. The aim of this study is to adjust the intervention and the logistics as well as to identify potential themes not yet integrated into the questionnaires. We will invite about 30 women to participate. Instead of following up with questionnaires at 6 weeks and 6 months post partum, a focus group interview will be performed 3–4 weeks post partum

**Power calculations**

The power calculations are based on the primary endpoint, use of epidural analgesia during childbirth. In 2007, 44% of all primiparas giving birth at Aarhus University Hospital Skejby received epidural analgesia. We presume that the women who want to take part in the project will have a lower frequency in the use of epidural analgesia, 40%, and that the use of epidural analgesia can be reduced to 35% by relaxation techniques, and 25% by self-hypnosis. On the basis of these assumptions, we must include 328 participants in the hypnosis group, 328 participants in the active comparator group, and 152 participants in the control group to obtain a power of 80% (α 0.05 (two-sided)) to detect a difference of 5 percentage points (RR: 0.71) between the intervention and the active comparator groups. By including 152 participants in the control group, we will have a larger power to detect a difference of 15 percentage points (RR: 0.63) in the use of epidural analgesia between the intervention and the control groups.

Because we expect that some participants will develop medical conditions that required epidural analgesia during delivery, give birth prior to receiving their allocated intervention, or give birth by caesarean section, we decide to increase the sample size by 10% in all groups to a total of 890 participants.

Using the estimated number of participants with regard to epidural blockade, a strength of 90% is needed to show a difference of 60 min in duration of labour of between the intervention and control groups. These calculations were performed based on an expected duration of labour of 480 min and 420 min in two groups of the same size, with a range of 220 min in both groups. On this basis, 283 participants will be included in each of these groups.

During the course of the study, an interim analysis will be undertaken after the first 12 months in order to make any necessary adjustments in number of participants

**Perspectives**

Preliminary results in the area appear promising and suggest a marked reduction in use of other forms of pain relief, operative procedures during labour, and the negative results of these procedures. If the results of the present studies follow the same tendency as the preliminary results, it can mean economic savings for the entire healthcare system.

**Safety**

In the metaanalysis that studied the effect of hypnosis on pain relief during labour, 19 studies were identified, and in none of them were there any reports of harmful effects of hypnosis. The conclusion is that there is very little basis for a fear of any harmful effects. [29]. This is supported by the Danish Knowledge and Research Centre for Alternative Medicine [41]. In this project, cooperation with highly educated and experienced professionals in the area is given priority. Intervention, the self-hypnosis programme, will be developed in dialog with Australian researchers who have used the technique for more than 10 years in the healthcare system [24]. To achieve the greatest possible safety, only mentally healthy women will be enrolled in the project. Women who attend the first session and then fail to appear will be contacted by telephone to determine the reason for their absence.

**Ethical considerations**

Participants in the project will be offered something more than what is already offered. On the basis of research and experience, the intervention is considered to not have any harmful side effects. Thus no one will be at any disadvantage with regard to the existing prevention and treatment regimens

On the contrary, there is the prospective to study new, effective techniques that can alleviate labour pain without side effects. If the results of the study show a beneficial effect of the studied technique, it can mean that personal and societal expenses will be reduced. The economic perspective is also important to consider in an ethical context, because the release of economic resources will mean that initiatives can be financed in other areas in the Danish healthcare system.

**Permissions**

The research was approved by the Danish Data Protection Agency and the Scientific Ethical Committee for the Region of Central Jutland. The trial will also be reported to ClinicalTrials.gov.

**Supplement to the protocol, 2009**

Before starting the trial, it was decided that the interim analysis would not be undertaken.

**Supplement to the protocol, 2010**

At the start of the study, we performed our power calculations based on the fact that 44% of all primiparas at Aarhus University Hospital Skejby in 2007 had received epidural analgesia. We hypothesised that the observed frequency of epidural analgesia would be 25% in the intervention group, 35% in the placebo effect group, and 40 % in the placebo group.

According to these power calculations, we should include 328 participants in the hypnosis group, 328 participants in the active comparator group, and 152 participants in the control group to obtain a power of 80% (α 0.05 (two-sided)) to detect a difference of 5 percentage points (RR: 0.71) between the intervention and the active comparator groups. By including 152 participants in the control group, we would have a larger power to detect a difference of 15 percentage points (RR: 0.63) in the use of epidural analgesia between the intervention and the control groups.

Because we expected that some participants would develop medical conditions that required epidural analgesia during delivery, give birth prior to receiving their allocated intervention, or give birth by caesarean section, we decided to increase the sample size by 10% in all groups to a total of 890 participants.

In 2010, the study board experienced that in 2009 the general frequency of epidural analgesia in primiparas giving birth at Aarhus University Hospital Skejby had decreased to 34%.Therefore it was decided to adjust the sample size according to this new information.

When the sample size was adjusted, we maintained the same sample ratios between the groups and hypothesised that the observed frequency of epidural analgesia would be 22 % in the intervention group, 30 % in the placebo effect group, and 32 % in the placebo group. To obtain a power of 80% (α 0.05 (two-sided)) in detecting a difference of 8 percentage points (RR: 0.73) between the intervention and the active comparator groups in the use of epidural analgesia and a difference of 10 percentage points (RR: 0.68) between the intervention and the control groups, we should include 446 participants in the hypnosis group, 446 participants in the active comparator group, and 226 participants in the control group, in total 1097 participants. Again, we further increased the sample size with an extra 10% to account for those women that would not be able to comply with their allocated treatment because of obstetric conditions, and reached a sample size of 1208.

**Appendix 1: Studies**  on hypnosis and childbirth

| **Study** | **Sample**  **N** | **Design** | **Intervention** | **Outcomes pain** |
| --- | --- | --- | --- | --- |
| Cyna et al  (2006) (24) | 77/3249  Australia | Case control  Self-selection | 3-4 x Individual sessions in self-hypnosis vs matched controls  Start: After 35 gw | Epidural analgesia |
| Cyna et al  (2004) (29) | 74/68 | Meta-analysis |  | Analgesic medication  RR 0,51 (95%CI; 0,28;0,95) |
| Mehl-Madonna  (2004) (34) | 250/250/(250)  USA | Randomised Controlled Trial  (a machted control group added in analysis) | Hypnosis vs Psychotherapy vs Paired control group  (vs matched control group)  Individual sessions  amount as needed, mean 3 times (Range 1- 60)  Start 1.or 2.trimester | In favor of hypnosis (HG/ PG/ PCG):  Any analgesic medication, (28%/67%/83%)  Epidural analgesia, (28%/67%/83%) |
| Martin et al  (2001) (36) | 22/20 | Randomised Controlled Trial | Hypnosis vs supportive counseling  Both groups 4 x  Hypnosis: Self-hypnosis Hypnoreflexlogymethod incl. information on labour and delivery  Start 20-24 gestational week | -Anaesthesia (45% vs 70%, NS)  -Pitocin (9% vs 30%, NS)  -Postpartum medication (9% vs 55%, NS) |
| Jenkins et al  (1993) (31) | 126/300  136/300 women  Wales | Case control | 6X ½ hour individual hypnosis vs matced controls | Analgesic medication |
| Harmon et al  (1990) (35) | 30/30  USA | Randomised Controlled Trial | Hypnosis vs traditional childbirth education  Training in groups of 15  Both groups 6 x 1 hour on week basis and tapes for homework:  Hypnosis group: Suggestions to ease childbirth  Control group: Progressive relaxation + breathing exercise  Start: End of 2.trimester | In favor of high h (vs low h):  -Use of tranquilizers, p<0.01  -Use of Narcotics, p<0.01 |
| Bran et al  (1987) (32) |  | Clinical study  Self-selection | 2 grupper  4 x hypnose opfulgt af selvhypnose på tape vs psykoprofylakse med åndedrætsøvelser, afspændingsteknik og besøg på fødeafdeling  Start: GW 28 |  |
| Freeman et al  (1986) (38) | Nulliparous women  29/36  United Kingdom | Randomised Controlled Trial | Hypnosis vs traditional childbirth education  Training in groups of 15  Both groups 6 x 1 hour on week basis and tapes for homework:  Hypnosis group: Received individual sessions on week basis from 32 g.a.week focused on relaxation + glove anesthesia  Start: End of 2.trimester | -Analgesic HG vs CG, NS  -High+moderat susceptible vs Poor: less use of epidural analgesia  p<0.01 |
| Guthrie et al  (1984) (42) | 8(8 women | Case control prospective |  | Self-rated pain |
| Rock et al  (1969) (37) | Parturient women  22/18  USA | Randomised Controlled Trial | Hypnosis during childbirth by a hypnotherapist (HG) vs Controls receiving continuous presence of a medical student (CG)  Start: At birth | -Use of analgesic (demetrol and a tranquilizer): HG:52% CG:76%, p<0.05  -Observed comfort: HG more comfortable than CG, p<0.05  -Self-rated pain: HG less pain than CG, p<0.01 |

1. Which also includes biofeedback, cognitive therapy, visualisation and meditation. [↑](#footnote-ref-2)
